# Comparison of Partec Rapid Malaria Test with Conventional Light Microscopy for Diagnosis of Malaria in Northwest Ethiopia

**DOI:** 10.1155/2016/3479457

**Published:** 2016-01-11

**Authors:** Meseret Birhanie

**Affiliations:** Department of Medical Parasitology, School of Biomedical and Laboratory Sciences, College of Medicine and Health Sciences, University of Gondar, P.O. Box 196, Gondar, Ethiopia

## Abstract

*Background*. Laboratory diagnosis of malaria is the key for effective disease management. Diagnosis of malaria infection requires rapid, sensitive, and specific test methods with an affordable cost. This study was aimed to assess the diagnostic performance of Partec rapid malaria test with reference to light microscopy for the diagnosis of malaria in Northwest Ethiopia.* Methods*. A total of 180 febrile patients were tested for malaria using Giemsa stain microscopy and Partec rapid malaria test from June to July 2013 at Gendewuha health centers, Metema district. Data were analyzed using SPSS version 20 statistical software. Odds ratio with 95% CI was calculated.* Result*. The sensitivity and specificity of Partec rapid malaria test were 93.8% (95% CI = 87.1%–100%) and 87.9% (95% CI = 79.7%–96.1%), respectively, while the positive predictive value and negative predictive value were 6.4% (95% CI = 77.2%–95.5%) and 94.6% (95% CI = 88.7%–100%), respectively. There was also an excellent agreement between two tests with Kappa value of 0.811 (95% CI = 0.625–0.996).* Conclusion*. Partec rapid malaria test showed good sensitivity and specificity with an excellent agreement to the reference light microscopy. Therefore PT can be considered as alternative diagnostic tools in malaria endemic areas.

## 1. Background

Malaria is the most common fatal disease in the world. According to 2013 WHO malaria report, an estimated 3.4 billion people were at risk of malaria globally and populations living in sub-Saharan Africa have the highest risk of acquiring malaria. Approximately 80% of cases and 90% of deaths are estimated to occur in the African region [1]. In Ethiopia approximately 52 million people (68%) live in malaria risk areas.* Plasmodium falciparum* and* P. vivax* are the dominant species of malaria in Ethiopia, with 60% and 40% relative frequencies, respectively.* P. falciparum* is the most dominant species in endemic areas and causes severe and complicated disease and death [2].

In Ethiopia half a million microscopically confirmed cases of malaria are reported to the Federal Ministry of Health (FMOH) in each year from basic health services. However, the actual number of malaria cases in the country is estimated to be more than 5 million each year [2]. So, choosing an accurate, rapid, and cost-effective diagnostic test is mainstay of efficient clinical and epidemiological management of malaria [3, 4].

Clinical diagnosis is the most widely used diagnostic method in rural areas and where laboratory facility does not exist. It is inexpensive to perform and requires no special equipment or supplies [2]. However, according to WHO 2011 guidelines clinical diagnosis of malaria based on signs and symptoms alone is not recommended since it has low specificity and increases the chances of the patient being misdiagnosed and leads to misuse of drugs [5, 6].

A laboratory diagnosis of malaria is one possibility in the management of a patient presenting with fever. To improve the quality care of the patients, many diagnostic procedures have been developed which aim to have accurate diagnosis, to reduce the time of preparation and training needed [3, 4]. Even though Giemsa stain microscopy remains a gold standard method for malaria diagnosis in many developing countries [4, 7, 8], but it is not 100% sensitive and specific [9].

The Partec rapid malaria test (PT) is newly innovative fluorescence microscopy method for the detection of* Plasmodium* species DNA in human blood. It uses readily prepared and ready-to-use slides labeled with an unspecific DNA-binding fluorescent dye (4′-6-diamidino-2-phenylindole (DAPI); emission 365 nm) that detects plasmodial DNA. It is ultra compact and robust microscope design and has connector for optionally available CCD camera upgrade for visualization of the slides on any PC Windows with USB connection [10, 11]. The sensitivity and specificity of the test were not assessed in Ethiopia. Thus this study was conducted to evaluate the performance of CyScope florescence microscopy (Partec rapid malaria test) in reference to light microscopy in Northwest Ethiopia.

## 2. Methods 

### 2.1. Study Area and Period

A study was conducted at Gendewuha health center found in Metema district, North Gondar Zone, Amhara Regional State, from June to July 2013. The district is found at the Ethio-Sudan border. It is far 925 km from Addis Ababa (capital city of Ethiopia) and found at 180 km west of Gondar town. The mean annual temperature ranges from 22°C to 28°C and the daily temperature reaches as high as 43°C during the months of March to May. Mean annual rainfall ranges from 850 mm to 1100 mm and has unimodal pattern. The altitudes range from 550 to 1068 meters above sea level [12]. The district is malarious and endemic for all* Plasmodium* species. But the prevalence of* P. falciparum *is very high. The health center is serving around 18,000 people in Gendewuha town and surrounding areas.

### 2.2. Study Subject

One hundred and eighty malaria suspected patients attending at Gendewuha health center during the study period were included and screened for malaria infection using light microscopy and Partec rapid malaria test. Patients who had received antimalarial drugs during the past two weeks, children under 1 year, and critically ill patients who were unable to respond to the interview were excluded from the study.

### 2.3. Specimen Collection and Processing

Sociodemographic and clinical data of the study participants were collected using structured questionnaire. Three milliliters of venous blood was collected with EDTA tubes by laboratory technicians from each study subject for smear preparation of light microscopy and PT.

### 2.4. Light Microscopy

After collecting blood samples, thick and thin blood smears were prepared and stained with 10% Giemsa working solution for 10 minutes. Blood films were observed under 100x objectives for the detection of malaria parasites and the result was reported as positive if asexual malaria parasites were seen or negative if malaria parasites were not seen after observing 100 fields of the thick smear. All blood films were reexamined by an experienced microscopist at University of Gondar Hospital laboratory who was blinded to initial light microscopy result.

### 2.5. Partec Rapid Malaria Test

**Figure 1:**
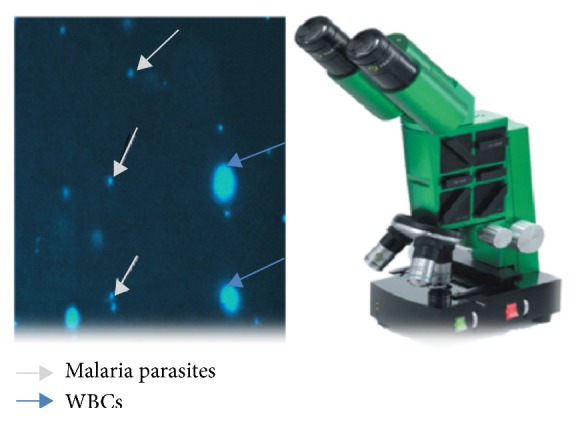
Partec CyScope fluorescence microscope and malaria parasites (adapted from [11]).

Partec rapid malaria test was carried out according to the manufacturer's instructions. Briefly, on the collected EDTA blood, 10 *μ*L blood was placed on the dye labeled area of a slide and cover slipped, incubated at room temperature for a minute, and observed with 40x objective under LED UV light (365 nm). The presence of bright shiny intracellular tiny dots against a dark background observed under the UV light indicated the presence of malaria parasites in the erythrocytes as shown in Figure 1.

### 2.6. Statistical Analysis

The data was entered into SPSS version 20 software for analysis. Odds ratio, sensitivity, specificity, positive predictive value, negative predictive value, and Kappa value were calculated at 95% CI.

### 2.7. Ethical Approval

Ethical clearance was obtained from University of Gondar, School of Biomedical and Laboratory Sciences Ethical Committee, and permission was obtained from Gendewuha health office and Gendewuha health center authorities to conduct the study. After informing about the objective of the study, written consent was taken from all study participants or parents/guardians. Participants who were positive to malaria parasite were given the appropriate treatment at the health centre.

## 3. Result

A total of 180 malaria suspected patients were diagnosed for malaria. About 99 (55%) of the study participants were males and 81 (45%) were females. The mean age of the participants was 19 ± 12.49 years and majority of the participants 60 (33.3%) were within the age range of 20–29 years, and 42.8% of the participants were illiterate. The rural residence has statistically significant association with malaria infection (AOR = 0.437, CI = 0.028–0.835) (Table 1).

### 3.1. Light Microscopy

The overall prevalence of malaria using light microscopy was 81 (45%). From this* Plasmodium falciparum* accounts for 76 (93.8%),* P. vivax *were 4 (4.9%), and mixed infection (*P. falciparum *and* P. vivax*) was 1 (1.3%). There were 2 discordant results between the first (study site) reader and the final reading by experienced microscopist; thus the final report was based on the final reading. Turnaround time of Giemsa stain microscopy was more than an hour.

### 3.2. Partec Rapid Malaria Test

The parasite positivity of malaria by using Partec rapid malaria test was 88 (48.9%). Seventy-six slides were positive with Partec rapid malaria test and the gold standard Giemsa stain microscopy. Twelve slides were positive with Partec rapid malaria test and negative with light microscopy, while 5 slides were negative with Partec rapid malaria test and positive with light microscopy and 87 slides were negative for both (Table 2).

### 3.3. Sensitivity and Specificity

Using light microscopy as standard test for diagnosing malaria, the sensitivity and specificity of Partec rapid malaria test were 93.8% (95% CI = 87.1–100) and 87.9% (95% CI = 79.7–96.1), respectively. There was also an excellent agreement between light microscopy and Partec rapid malaria test with Kappa value of 0.811 (95% CI = 0.625–0.996) (Table 3).

In addition, operational characteristics were observed. Compared to light microscopy PT is more sensitive, less labor intensive, and faster to use and has a less turnaround time than the LM (an average of 5 minutes). PT also requires very little training and there is no need of reagent preparation. Furthermore, the CyScope florescence microscope is battery operated; the battery can be kept on for 6 hours after full charge making it best for field work. It is easier to use and to adjust and can be switched from UV light to bright field. However, the PT is not suitable for species identification and parasitic load determination and blood films cannot be stored for a long period of time.

## 4. Discussion

The current study revealed a high sensitivity and specificity of Partec rapid malaria test. The high sensitivity and specificity are in agreement with the reports in Sudan [13]. However, the sensitivity and specificity are lower than the reports in Zimbabwe [14], Ghana [11, 15], Sudan [16], and Uganda [17]. These differences might be due to observer variation or host factors. Partec rapid malaria test had high PPV and NPV. Thus, a high NPV indicates that a person does not have the disease with high certainty, meaning that PT is reliable test method in diagnosing of malaria parasites.

Partec rapid malaria test had detected 12 cases which were negative by light microscopy. Unlike Giemsa stain light microscopy, PT has sensitive fluorescent dye, 4,6-diamidino-2-phenylindole (DAPI), which can be detected in such low level of parasites. In addition, the presence of artifacts such as nonspecific aggregated DAPI, immature erythrocytes, or bacterial cells might have been misinterpreted as* Plasmodium* DNA [15]. On the other hand, PT produced 5 false negative results which were positive by light microscopy. This might be due to the fact that unlysed red blood cells may lie on each other or overlap with each other thereby preventing parasites in red blood cells that may be lying beneath other cells. The performance characteristics of the tests were very similar as indicated in their sensitivities, specificities, PPV, and NPV with very good agreements to the light microscopy.

Determination of parasitemia with Partec rapid malaria test is difficult, because the red blood cell may overlap each other and make the parasites invisible under the UV light. This finding is in line with the finding of the study conducted in Ghana [15]. Though, PT is useful as light microscopy thick film for screening purpose in malaria endemic areas.

The overall prevalence of malaria in the study area was very high, as detected by both the LM (45%) and the PT (48.9%). This result is higher than the report from other regions in Ethiopia [18, 19]. The high prevalence could be partly explained by the fact that the study was conducted in malaria transmission season of the country.

## 5. Conclusion

Partec rapid malaria test showed good sensitivity and specificity with an excellent agreement to the reference light microscopy. PT has very short turnaround times, requires little training, and is applicable under field conditions. Therefore PT can be considered as alternative diagnostic tools in malaria endemic areas. Further studies are needed to determine the cost-effectiveness and long term performance of the Partec rapid malaria test in diagnosing malaria.

## Figures and Tables

**Table 1:** Sociodemographic characteristics of patients and malaria positivity by light microscopy Gendewuha health center, Northwest Ethiopia, from June to July, 2013.

Characteristics	Light microscopy						
	Tested patients *N* (%)	Positive *N* (%)	Negative *N* (%)	COR	95% CI	AOR	95% CI
Gender							
Male	99 (55.0)	53 (53.5)	46 (46.5)	0.459	0.250–0.839		
Female	81 (45.0)	28 (34.6)	53 (65.4)	1			
Age in years							
1–9	51 (28.3)	15 (29.4)	36 (70.6)	1.029	0.234–4.521		
10–19	44 (24.4)	17 (38.6)	27 (61.4)	0.681	0.155–2.997		
20–29	60 (33.3)	42 (70.0)	18 (30.0)	0.199	0.046–0.853	0.179	0.040–0.792
30–39	15 (8.3)	5 (33.3)	10 (66.7)	0.857	0.152–4.819		
≥40	10 (5.6)	3 (30.0)	7 (70.0)	1			
Residence							
Rural	86 (47.8)	49 (57.0)	37 (43.0)	0.390	0.213–0.713	0.437	0.0228–0.835
Urban	94 (52.2)	32 (34.0)	62 (66.0)	1			
Educational background							
Illiterate	76 (42.2)	39 (51.3)	37 (48.7)	0.632	0.100–4.00		
Read and write	39 (21.7)	17 (43.6)	22 (56.4)	0.863	0.129–5.756		
Primary school	51 (28.3)	21 (41.2)	30 (58.8)	1.033	0.158–6.741		
Secondary	9 (5.0)	3 (33.3)	6 (66.7)	1.333	0.139–12.818		
College/university	5 (2.8)	2 (40.0)	3 (60.0)	1			
Occupation							
Farmer	39 (21.7)	18 (46.2)	21 (53.8)	0.648	0.184–2.28		
Merchant	34 (18.9)	10 (29.4)	24 (70.6)	1.33	0.357–4.98		
Civil servant	7 (3.9)	2 (28.6)	5 (71.4)	1.389	0.194–9.96		
Daily laborers	47 (26.1)	31 (66.0)	16 (34.0)	0.287	0.082–0.99		
Housewife	30 (16.7)	12 (40.0)	18 (60.0)	0.833	0.224–3.10		
Private	9 (5.0)	4 (44.4)	5 (55.6)	0.694	0.126–3.83		
Student	14 (7.8)	5 (35.7)	9 (64.3)	1			

*N* = number; COR = crude odds ratio; AOR = adjusted odds ratio; CI = confidence interval.

**Table 2:** Results of light microscopy and Partec rapid malaria test.

	Light microscopy			
		Positive	Negative	Total
Partec rapid malaria test	Positive	76	12	88
	Negative	5	87	92
	Total	81	99	180

**Table 3:** Performance of Partec rapid malaria test using light microscopy as a standard.

Test	Sensitivity% (95% CI)	Specificity% (95% CI)	PPV% (95% CI)	NPV% (95% CI)	Kappa value% (95% CI)
Partec rapid malaria test	93.8% (87.1–100)	87.9% (79.7–96.1)	86.4% (77.2–95.5)	94.6% (88.7–100)	81.1% (62.5–99.6)

## Abbreviations

PT: Partec rapid malaria test
LM: Light microscopy
PPV: Positive predictive value
NPV: Negative predictive value
UV: Ultraviolet.
